# Principles, Approaches and Challenges of Applying Big Data in Safety Psychology Research

**DOI:** 10.3389/fpsyg.2019.01596

**Published:** 2019-07-09

**Authors:** Liangguo Kang, Chao Wu, Bing Wang

**Affiliations:** ^1^School of Resources and Safety Engineering, Central South University, Changsha, China; ^2^Safety & Security Theory Innovation and Promotion Center, Central South University, Changsha, China

**Keywords:** big data, multidisciplinary psychology, big data of safety psychology, organizational psychology, industrial safety

## Abstract

Big data is now widely used in many fields and is also widely applied to the integration of disciplines. Traditional methods of safety psychology are not well suited for analyzing psychological states, especially in the management of human factors in industrial production. Also, big data now becomes a new way to excavate related insight by analyzing a large amount of psychological data. So, this paper is to propose the concept of big data of safety psychology (BDSP) and to illustrate the challenges of applying big data in safety psychology. First, this paper puts forward the concept of BDSP and analyzes the difference between BDSP and traditional sample data. Subsequently, this paper summarizes the classification standard and basic characteristic of BDSP, explores the framework of BDSP and then constructs a three-dimensional structure of BDSP. Lastly, this paper discusses the challenges of using BDSP. This study is of great help to safety practitioners to solve psychological issues in the safety domain, and points out one of the research trends of human factor in industrial safety.

## Introduction

Psychology plays an important role in work safety (Cree and Kelloway, 1997). In the long period of productive practice, safety psychology became a subject of improving safety consciousness of workers in China. With the emergence of big data, disruptive technologies have transformed commerce, science and many aspects of society (Lin, 2015), evidently when *Nature* and *Science* published special issues dedicating to discuss the opportunities and challenges brought by big data. It marks the possibility that we can apply big data in various fields and reveals that thinking of data processing is correlation rather than causation. Meanwhile, there was a link between the psychological state of people and the tendency of behavior. Once the patterns of human acts (e.g., data record of text, images, and video) are analyzed by big data technology, personal psychological state can be inferred (Huyghebaert et al., 2017). Accordingly, we can apply big data to solve psychological issues related to safety. The aim is to analyze the correlation between psychological data and workplace risk, which provides valuable information of psychology for users (e.g., government, enterprises, and employees) to reduce the risk of the working environment.

Big data practitioners in academia, industry and community have built a comprehensive base of tools and knowledge that makes big data accessible to researchers in a broad range of fields (Chen and Wojcik, 2016). Thus, big data offer fresh ways to perfect and extend theoriesof safety psychology. Many studies analyze safety big data from perspectives of concepts, models and empirical analysis. For example, Ouyang et al. (2018) put forward the methodologies, principles and prospects of applying big data in safety science research, Huang et al. (2018b) constructed a conceptual framework of big-data-driven safety decision-making, and Marvin et al. (2017) explained a few examples of the new developments that big data was contributing to in early warning systems in food safety problems. In addition, crisis-related incidents generate data in the course of practice, especially when joined with data originating from other sources, create giant psychological related datasets (Guzzo et al., 2015). For one thing, job insecurity leads to stressful workplace conditions, which decrease people’s psychological well-being and mental health, and are even linked to the development of mental illness (Giorgi et al., 2016; Mucci et al., 2016). For another, psychosocial risk management during times of crisis is a strategic topic because crisis may be accompanied by an increase in mortality (Giorgi et al., 2015). As psychological data sets grow, they become increasingly valuable for safety policymakers to interfere with collective climate of fear and develop optimism in the workplace.

It is important to make an effective safety decision based on reliable and enough psychological related information in safety management (Wang et al., 2017). Various scholars have access to the massive quantities of information produced by people, things and their interactions in the era of big data (Boyd and Crawford, 2012). Thus, big data provides credible evidence based on psychological information analyzing, forecasting and managing people’s behavior. However, big data technology is not mature enough and thus leads to some data dilemmas, such as noise accumulation, spurious correlation, and incidental homogeneity (Fan et al., 2014). Also, another difficulty is how to manage the large quantity of psychological data efficiently and address the shortcomings of data in the current technological environment, such as semantic analysis, multimode data reduction, data plugging, data separation, slow data, and data missing. Despite the fact that there are still some problems, it is a fascinating outlook that unsafe psychology of human was intervened by evidence of safety information based on psychological knowledge and rules.

## The Proposal of BDSP and Its Concept

### The Defect of Traditional Safety Psychology

In China, safety psychology is almost a compulsory course for safety engineering students. The aim of safety psychology is that it can infer the psychological state by analyzing behavior, then explain, predict and intervene the human’s act, thus improve the human’s ability to handle risk factors in enterprises. Sample surveys of traditional safety psychology are a main way to discover the psychological knowledge and rule in the safety domain, including in the research methods of observation, survey, test, experimental and case analysis, as well as the research tool of psychological questionnaires and psychological scales (Zhu et al., 2015). However, the volume of study objects is limited, with it difficult to obtain thousands or more. The selection of study objects should meet the essential qualification of statistics, namely that samples selected are random with equal chances. As such, sample volume can reflect the situation of the entire study object.

Some psychological factors are often ignored in the entire research due to the difference in education, culture, environment, cognition, and work experience of study object (Fisher et al., 2017), as well as the limitations of sample volume and questionnaire design. The repeatability and reliability of research results are relatively low, which presents difficulties to large-scale promotion and application in the field of industrial safety. Since traditional methods of safety psychology can’t meet the complex requirement of safety activities, big data applied to safety psychology research in such a context is nurtured and hygienic, providing safety managers with evidence-based services of behavior management and task disassembling.

With the era of big data coming, especially when Internet companies have accumulated the application and optimization experience of data mining in the long run, it has achieved the objectives in term of automatic and real-time storage, data processing and analyzing, and personalized service provided according to user’s psychological characteristics and behavioral habits. For example, e-commerce companies send messages to his customers about various products likely to interest him to the app homepage according to the user’s psychological characteristics indicated by online shopping history.

### Scholars Use Big Data to Extract Psychological Knowledge

Big data is a well-known data processing technology in use today which has been employed in various fields. With the view that the background for the current study circumstances is the cross-integration trend of the subject area, many researchers and practitioners use big data technology to explore and verify psychological issues in recent years as shown in Table 1. Accordingly, new psychological knowledge and rules were excavated by big data, are also applied to address the challenge faced by traditional safety psychology.

**Table 1:** Brief review of psychological knowledge discovered by big data.

Authors	Psychological knowledge
Golder and Macy, 2011	509 million messages authored between February 2008 and January 2010 for 2.4 million individuals from the globe were analyzed. The results showed that (i) Positive effect had two peaks: relatively early in the morning and again near midnight. (ii) A morning rise and nighttime peak in positive effect, and a sharp drop in negative effect during the overnight hours. (iii) Higher positive effect on the weekend than during the work week, and a delayed weekend of nearly 2 h.
Curini et al., 2015	Over 43 million tweets posted on a daily basis in all of the 110 Italian provinces during 2012 were analyzed. The results showed that the determinants of happiness were meteorological variables and events related to specific days. For example, an increase of temperature in winter and spring augments iHappy significantly, and a 1°C increase in temperature translates into a half point augmentation of iHappy, with 30°C as the upper limit in summer.
Wang et al., 2016	1.95 million active users of Weibo, a Chinese social platform like Tweet, from September 2011 to June 2012 were analyzed. The results showed that (i) Positive mood change peak at 1 p.m. and 8 p.m., and negative mood change peak at 3 a.m. (ii) Negative effect on weekends was lower than that on weekdays. (iii) Positive affect and negative affect were the highest in autumn and the lowest in summer.
Jones et al., 2016	Longitudinal Twitter data across 3 case studies were analyzed to examine the impact of violence. The results showed that both communities users and campuses users, negative emotions performed were higher within 6 weeks after the incident, and negative emotions performed most significantly in a week, then returned to the benchmark level after 2 weeks.
Ye et al., 2016	94,562 valid posts about the moral emotions expressed toward the “7.23 Wenzhou Train Collision” by Chinese netizens on Weibo were analyzed. The results showed that men were more likely to express anger, disgust and contempt with higher intensity while women were more likely to express compassion and love.
Vaitla et al., 2017	Big data are most powerfully used to supplement traditional experimental paradigms in order to understand human behavior and cognition.
Adjerid and Kelley, 2018	Big data research efforts are more readily accessible than many researchers have realized, and opportunities for big data research are diverse and differ a lot, and using big data and benefiting from it in psychology is cautiously optimistic.

### The Proposal of BDSP

Interdisciplinary research is one of the popular research fields in organizational psychology, as is also determined by the characteristic of psychological issues in industrial safety. It is necessary to the disciplinary intercrossing and fusion of safety science, data science and psychology, which solve the psychological issue in the safety domain from theoretical innovation and practical application.

Data science is a discipline using data to learn knowledge, including applied mathematics, pattern recognition, machine learning, data warehouse and high-performance computing. One of its aims is to reveal the phenomena and laws of human behaviors. The emergence of big data has spawned a new research paradigm and provided a bright prospect to numerous disciplines. Hence, we can use big data to address safety issues.

Safety psychology is the intersection of the disciplines of safety science and psychology. Basically, the knowledge and skills from safety psychology need to be mastered by safety practitioners, which is the result of long-term application and practice in the enterprise. Also, safety psychology is a mature discipline in China. With the rapid development of information technologies, a large amount of data have been produced for almost every aspect of people’s lives and work, and these data can be used to solve safety problems. Big data applies in the safety domain to make a large amount of theoretical innovation and expansion, including safety big data proposal (Ouyang et al., 2018), accidents analysis paradigm (Huang et al., 2018a), and big-data-driven safety decision-making (Huang et al., 2018b). Big data can also be used in coal mine safety (Abou El-Nasr and Shaban, 2015), construction safety (Guo et al., 2016), traffic safety (Shi and Abdel-Aty, 2015), and food safety (Wang et al., 2015). Furthermore, the massive quantities of data covering human behaviors and moods offer psychology an unprecedented opportunity to conduct innovative theory field study (Chen and Wojcik, 2016). Applying big data in psychology will also gradually mature, and many scholars have carried out lots of theoretical studies (Cheung and Jak, 2016; Harlow and Oswald, 2016; Adjerid and Kelley, 2018) and practice studies (Dhar, 2013; Vie et al., 2013; Paxton and Griffiths, 2017; Leivada et al., 2019).

**FIGURE 1:**
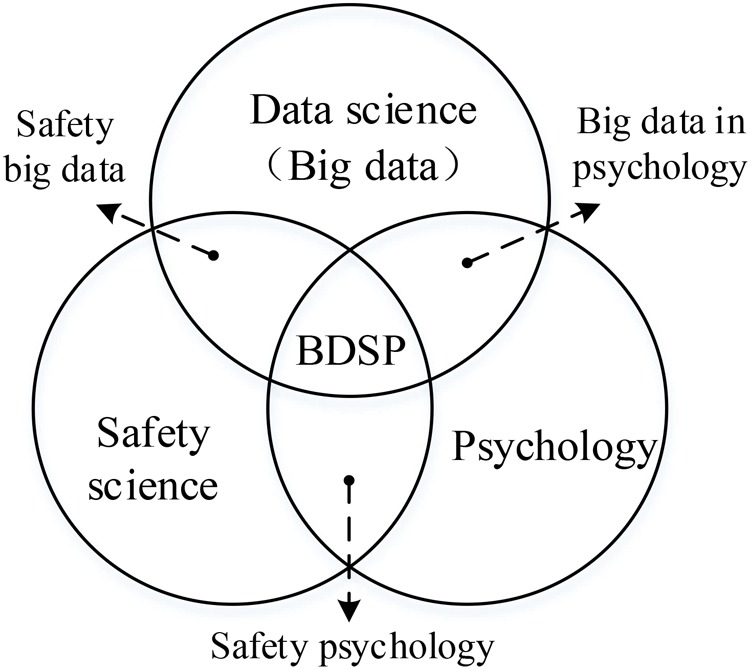
Discipline intersection* of safety science, data science, and psychology.

According to what has been discussed above, safety science, data science and psychology are no longer independent but linked to each other (e.g., safety psychology, safety big data, big data in psychology), which has developed into a new disciplinary field, namely big data of safety psychology (BDSP), as shown in Figure 1. Also, a basic definition of BDSP is described according to the content of safety science, psychology, and big data.

Big data of safety psychology refers to structured, semi-structured and unstructured data set formed by psychological index parameters and behavior, which provide potential and valuable psychological knowledge and rules to solve the psychological issue related to safety with the help of big data technology.

The concepts of BDSP are further analyzed as follows:

(1) Data volume is beyond the maximum handling capability of the traditional computational technology within a given time period (Manyika et al., 2011), and the computing model and storage pattern of current computers levels can’t meet data analyzing requirements, thus it shall depend on distributed processing, distributed database, cloud storage, and virtualization technology based on cloud computing.
(2) BDSP is a set of various psychological data, including structured data (e.g., number, symbol), semi-structured data (e.g., XML document, HTML document) and unstructured data (e.g., text, image, video, and sound). The data types are complex, and the value density of information is quite low.
(3) BDSP does not mean a large amount of psychological related data in the safety domain, which can provide valuable psychological information to solve the psychological issue related to safety. In addition, the mining results need to satisfy the actual application requirement of the current permit condition of big data mining cost and information value density.

The aims of BDSP are as follows:

(1) The relationship between psychological factors (e.g., mood, ability, personality, gender, age, experience, and environment) and workplace risk were analyzed by a large amount of multi-modal data and collected accident cases.
(2) Human’s behavior simulations in the early, middle and late stages of the accident were performed by big data technology, which constantly optimized personal mode of psychological cognition and reactions, and then provided evidence-based information for the improvement and development of 3E countermeasures (e.g., engineering, education, and enforcement).
(3) It can contribute to reducing safety psychological information asymmetry among community, industry, enterprise and individual level, sequentially exerts an imperceptible influence on people’s safety consciousness.

### The Difference Between BDSP and Traditional Sample Data

Through literature analysis, it is not hard to find out that big data has plenty of differences from sample data (Ouyang et al., 2018). Differences from 12 aspects between BDSP and traditional sample data were analyzed briefly (Kang et al., 2017a), as shown in Table 2. Both BDSP and traditional sample data have advantages and disadvantages in different situations. Rationally choosing corresponding research methods is a scientific approach to solve psychological issues in the safety domain. Also, the purposes of both are to find knowledge and rules and further to reduce accidents and to create a better working environment.

**Table 2:** Differences between BDSP and traditional sample data.

Difference point	BDSP	Traditional sample data
Data sources	Data sources are diverse, including wearable device, smart device, internet trace data, and safety activity behavior record. Also, electronic data are real-time transmission and interaction with the clouds.	Data sources are relatively single, mainly obtained from the data collected by the safety psychological experiment or questionnaire survey, then the manual screening of valuable information.
Data variety	Unstructured data are dominant, including text data, multimedia data and behavioral data, which are related to the psychological condition. So, data varieties are complex.	Structured data are dominant, including cross-sectional data sets, longitudinal data sets, and panel data sets. So, data varieties are relatively simple.
Data volume	The volume of data is huge, exceeding the processing capability of traditional software tools, thus cloud computing is needed.	The volume of data is small, and data collected are mostly among a scale of hundreds of people which can be handled by traditional software tools.
Data values	Data include the factors related or unrelated to psychological states, all of which need to preprocessing and be converted into digital information. So, data density is uneven and data value is low in general.	Emphasis is on result data, and the unusual process data have been filtered out. So, data content is clear and data value is high in general.
Data relationships	The data relationship is usually unknown and has interference of noisy data. The factor correlation can be inferred by data preprocessing and mining.	Safety psychological experiment and survey is verification of study hypothesis, so, data collected more or less a simple or complex correlation.
Study requirements	Data are obtained from the whole study object, which needs to comply with the basic characteristics of statistics and big data.	Samples selected are random with equal chances, and sample volume can reflect the basic situation of the entire study object.
Study cycles	The overall study cycles are relativity short when combined with mature data mining algorithms and clouds computing.	Parameters design and samples selected stages are slow, and data analysis stages is fast. The overall study cycle is long, which may be last for several months or years.
Study costs	Separately building systems of data cleaning, data mining and clouds computing are often expensive. However, the costs are relativity cheap when renting a specialized big data service system of safety psychology.	The investment is different according to safety psychological sample volume and experimental environment parameters. Overall, the costs are at a moderate level.
Study logic	First, capture the streams of big data, then, discover psychological phenomena and rules with big data mining technology, lastly, analyze mining results, and reach some conclusions.	First, put forward the study hypothesis, then, design the study protocols according to the hypothesis, and acquire study results, lastly, accept or reject the hypothesis according to results analysis.
Study method	Emphasis is on data mining algorithms, such as classification analysis, affinity grouping or association rules, and cluster analysis.	Emphasis is on the design of experimental planning, parameters, survey and statistic, such as survey method, test method and experimental method.
Study technology	Emphasis is on recording behavioral data of study objects without interference with normal activities, such as wearables, smart devices and virtual reality.	Emphasis is on traditional artificial experimental techniques, social survey and statistic.
Study conclusion	The reliability of the conclusion is relatively high and extensively influential. The conclusions can instantly guide safety activities and provide warnings and intervening information.	The reliability of the conclusion is relatively low and narrowly influential. The conclusions should be repeatedly tested and refined.

## The Type and Characteristic of BDSP

### The Type of BDSP

Managing a large amount of psychological data is complex system engineering. Besides, BDSP are studied from different perspectives, so that twice as much can be accomplished with half the effort. The classification of BDSP help better understand its study content and development trend, which could be targeted to solve psychological problems and avoid detours (Ouyang et al., 2018). The types of BDSP are divided into seven categories, as shown in Table 3. It is noteworthy that the classification is not immutable but may change accordingly with the development of safety science, psychology, and big data.

**Table 3:** Types of BDSP.

Classification standard	Classification item	Explanations and examples
Psychological structure	Implicit BDSP	Implicit BDSP refer to the data sets that directly reflect safety psychological states of the worker, mainly composed of structured data, for example, blood pressure, heart rate, response time, fatigue, sensory, and other parameter data.
	Explicit BDSP	Explicit BDSP refer to the data sets that indirectly reflect safety psychological states of the worker, mainly composed of unstructured data, for example, behavioral photos, workplace video, and conversation audio.
Study object	Individual BDSP	Individual BDSP refer to the data sets related to individual psychological states, such as individual data of act, facial expressions and psychological indicators.
	Group BDSP	Group BDSP refer to the data sets related to group psychological states, such as job group data of blasters, transporters and miners.
	Industrial BDSP	Industrial BDSP refer to the data sets related to industrial psychological states, such as industrial data of chemical and mining industry.
Study area	Theatrical BDSP	Theatrical BDSP refer to the theoretical basis for the principles, laws, characteristics and methods of BDSP, such as the concept and rule of BDSP.
	Application BDSP	Application BDSP refer to the applied activity for the interpretation, prediction, intervention and management of BDSP, such as enterprise optimize employee work schedules based on excavating psychological relevance insights.
Study content	Factor-related BDSP	Factor-related BDSP refer to the data sets that can reveal the relationship between psychological factors (e.g., age, personality, experience) and the risk, for example, a large quantity of data in Twitter are analyzed to conclude the knowledge and rule of fatigue, mood, and attention, which can be applied in safety activities to interfere collective climate of unsafety.
	Countermeasures-related BDSP	Countermeasures-related BDSP refer to the data sets that can reveal psychological countermeasure prevent the risk. For example, machine design needs to analyze a large amount of data to satisfy the requirement of engineering psychology.
Update speed	Static BDSP	Raw data of static BDSP are updated slowly, such as psychological data manually collected by companies, institutions and organizations.
	Dynamic BDSP	Raw data of dynamic BDSP are updated real-time, such as automatic psychological data acquisition from sensor, Internet and smart device.
System boundary	Self-system BDSP	Self-system BDSP refer to the data sets that are obtained from psychological activities and behaviors of the individuals and groups, such as assessment data of employee psychological state.
	Other-system BDSP	Other-system BDSP refer to the data sets that are obtained from the impact external environment on psychological states, such as temperature, humidity, temperature and lighting.
Risk situation	High-risk BDSP	High-risk BDSP refer to the data sets that are obtained from industries with high-risk factors or high accident rate (e.g., mining industry, construction industry and chemical industry), which excavating psychological relevance insights for high-risk industry.
	Non-high-risk BDSP	Non-high-risk BDSP refer to the data sets that are obtained from industries with non-high-risk factors or relatively low accident rate (e.g., manufacturing industry and service industry), which excavating psychological relevance insights for non-high-risk industry.

### The Basic Characteristics of BDSP

**FIGURE 2:**
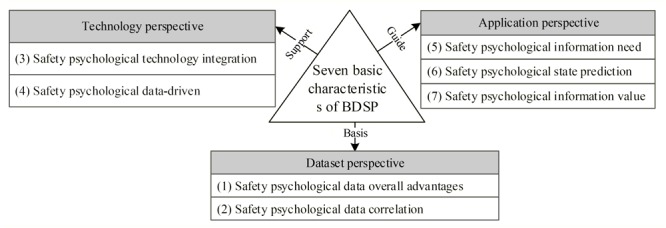
Basic characteristics of BDSP.

The goal of characteristics can be viewed as a deeper understanding of BDSP application and a guidance of how to apply BDSP appropriately. Data collected has hidden information of psychological states of people, which may be of little value to safety practitioners without big data mining technology. Thus, an interdisciplinary approach is needed to deal with psychological problems in industrial safety (Huang et al., 2018b). BDSP is the development trend for information technology applied to psychology. The application value of big data comes from three sources, according to three elements of big data (Mayer-Schönberger and Cukier, 2013). On this basis, the dataset serves as a basis, technology as a support, and application as a guide. Therefore, seven basic characteristics can be concluded, as Figure 2 shows. To further clarify the connotations of each characteristic, more detailed explanations can be seen as follows.

(1) Safety psychological data overall advantages. In the area of big data, psychological data are not obtained from samples selected randomly, but covers the whole study object, excavated from the underlying psychological knowledge and rules to improve the safety climate of organizations well. In addition, the traditional psychology study has been simplified as several important factors to analyze the relationship between behavior and psychological states. It is sometimes difficult to find the latent psychological rules from the heavy industry, light industry, and agriculture industry due to the limited study object volume. By contrast, related insight can be excavated from a large amount of psychological data since BDSP cover the whole study object. Meanwhile, the whole data produced by simple algorithms are more accurate than the sample data produced by complex algorithms (Halevy et al., 2009; Sacristán and Dilla, 2015). In practice, we should set a fault-tolerant standard of psychological data and quickly gain general outlines of psychological knowledge related to safety. For example, in 2015, occupations in China were divided into 1481 types, leading to the difficulty of recognizing the characteristics of each occupation by using traditional methods. But, it is quick to excavate the general rules of those occupations according to a large amount of psychological data in safety activities.
(2) Safety psychological data correlation. The correlation of psychological data is the core of BDSP (Ouyang et al., 2018), therefore many data mining methods have been emerging. Correlation thinking is a prior logic way in which the relevant insight is excavated from the stream of big data. Also, the major advantages of correlation study are to explore the unknown knowledge and rules related to safety psychology, to broaden the study areas of safety psychology, then to check the reliability of traditional sample results. For example, Golder and Macy (2011) applied big data to psychology to excavate the correlation of data acquisition where emotional information more than 500 million Twitter data from approximately 2.4 million users in 84 countries from February 2008 to January 2010. The results showed a volatility pattern of positive emotions and negative emotions.
(3) Safety psychological technology integration. The formation of BDSP benefits from the integration of cutting-edge information technology, and its application relies on the information technology level currently. It is a critical component of how to solve the technological problem of massive scale auto-implement of data acquisition, storage, processing, and visualization. So far, capturing the streams of big data in psychology mainly concentrates on Internet behavior records (e.g., Twitter, Weibo), which has achieved some success in psychological knowledge and rules. Real-time record and trace psychological data can be applied in a large scale in industrial production with the evolution of information technology.
(4) Safety psychological data-driven. Data-driven has become a popular and promising study method, incorporating almost all disciplines (Huang et al., 2018b). It excavates information value from a large amount of data, and integrates and refines the rules of information, thus forming an automated decision-making model. The system automatically makes decisions according to the previously established model without manual assistance, when raw psychological data is input. The bias information from the model analysis between the data-driven results and the actual results will give feedback to machine learning, and the model will self-improve in the subsequent machine learning iterative process. The complete data-driven value system is constructed from the data acquisition, management and mining, which play an important role in BDSP.
(5) Safety psychological information need. Due to the sectors and scales of enterprises differences, they have a practical need in the type and direction of psychological information, especially in high-frequency accidents industry and near-zero accidents service industry. Enterprises choose the level of big data mining technology according to its own safety condition, which intervenes the unsafe climate of formal and informal group. The real-time processing and analyzing of psychological data should satisfy the need of psychological information critical in safety evidence-based decisions. Therefore, the need for psychological information in the safety domain is the driving force for the technological innovation and development of BDSP.
(6) Safety psychological state prediction. With the development and popularity of wearables, psychological data be tracked and recorded without interfering with the activity of the worker, with electronic data automatically acquired, storage, and updated by smart devices, memory technology, and communication technology. According to the models (e.g., active learning, semi-supervised learning, transfer learning, and multitask learning) and algorithms (e.g., Apriori, C4.5, and AdaBoost) of big data, users instantly predict the psychological condition of individual, group and even the industry when combining data with a visual information system, providing practical evidence for safety decision-making and management. For example, Candás et al. (2014) used the data mining technology to predict people with mental disorders according to personal data gathered from wearables.
(7) Safety psychological information value. BDSP does not mean that a large amount of psychological data is related to safety. Its core is that psychological data can be transformed into valuable information for safety practitioners. Because the psychological state of workers is difficult to quantify, it requires managers to have relatively reliable psychological information to prevent workplace risk. Enterprises selects proper methods and tools to excavate the psychological rule to meet information value requirements in practical product. For example, the system real-time monitoring and recording information of miners’ psychological states by wearables, and they further correlate with storage data, which automatically alert miners of the attention against danger.

## The Framework and Structure of BDSP

### The Framework of BDSP

**FIGURE 3:**
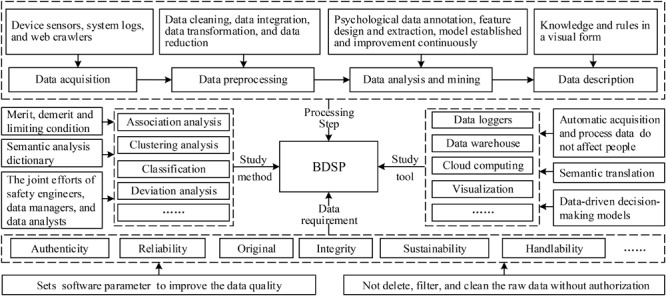
Framework of BDSP.

A reasonable framework of research will be critical to master BDSP. The framework of BDSP will standardize and guide safety psychological application in the big data age. From the perspective of data requirement, study method, study tool and processing step, they have formed the framework of BDSP, as shown in Figure 3. To further clarify the framework of BDSP, more detailed explanations can be seen as follows.

#### Data Requirement

Data acquisition is the basic condition for big data technology to study the psychological issues in the safety domain. Data acquisition methods are diverse and data quality is closely related to data sources, and on this basis, data acquisition tools are constantly emerging accordingly. Web crawling is one of the widely used ways for data acquisition automatically in recent years. The user sets the crawler software parameter to improve the data quality, ensuring the authenticity, reliability, originality and integrity of the raw data acquisition. Data management is performed, such as multimodality data management, text data processing and analysis, and feature system design and extraction, which come to some reliable conclusions. The user cannot delete, filter and clean the raw data without authorization to avoid error or bias.

#### Study Method

Data mining aims to reveal hidden, unknown and potentially valuable information from a large quantity of psychologically related data. According to the cost of using data mining tools and the fault-tolerant standard of psychological data, the data mining methods includes classification, regression analysis, clustering analysis, association rules, feature analysis, variance analysis and web page mining. Also, the data mining algorithm includes neural networks, genetic algorithm, decision tree and fuzzy set. Every method has its own merits, demerits and limiting conditions. Data collected have contained information related to the temperament, characteristics and emotions of personnel psychological characteristics, which need to establish semantic analysis dictionary to explain the connections between psychological data and the risks. Selecting and optimizing data mining methods need the joint efforts of the safety practitioners, data managers and data analysts, thus extracting the hidden and valuable psychological knowledge from the stream of big data will be faster and more efficient.

#### Study Tool

Psychological related data acquisition and processing has become more convenient and intelligent as devices are tending to be smaller and smarter. Also, the development of information technology and its own software and hardware component gradually mature. Study tools of safety psychology have changed from data acquisition to results visualization, especially behavioral data acquisition of study objects at the individual and group level. With the arrival of the era of big data, it has reached the basic condition in analysis visualizations, data mining, predictive analytic, semantic search, data management and data storage. The data logging from wearables, smart devices and virtual reality automatically store in a data warehouse to improve the authenticity and accuracy of raw data, which interact with the cloud in the form of electronic data transmission to form data-driven decision-making models, then the results have displayed in visualization ways.

#### Processing Step

The rising complexity of safety psychological problems means it is now imperative that we take scientific and rigorous steps to ensure BDSP can be better applied to people’s lives and work. On the basis of the step of big data mining and the characteristic of safety psychology, the models of BDSP were concluded as follows: (i) Data acquisition. Data acquisition is the application foundation of big data and its methods are now include three categories: device sensors, system logs and web crawlers. (ii) Data preprocessing. We need to take preprocessed methods due to data imperfection of missing, repetition, noise, inconsistency and high dimension, including data cleaning, data integration, data transformation and data reduction. (iii) Data analysis and mining. This step is a key of big data processing which can be simplified into psychological data annotation, feature design and extraction, safety psychological model established and model improvement continuous. (iv) Data description. This step is the description of the psychological knowledge and rule from a large amount of data in a visual form, which enables users to observe and analyze the results more efficiently.

### The Hierarchical Structure of BDSP

**FIGURE 4:**
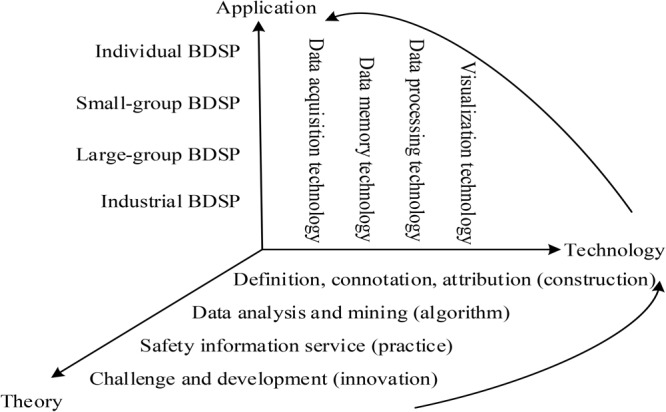
Three-dimensional structure of BDSP.

Application of BDSP is a complex activity which integrates theory level, information technology and application range into an organic whole. On this basis, we attempt to construct a three-dimensional structure of BDSP, as shown in Figure 4. To further clarify the level of BDSP, more detailed explanations can be seen as follows.

#### Theory Level

The promotion, development, dissemination and application of BDSP are the theoretical cornerstones, including the application characteristics of safety psychology and the technological superiority of big data. The theoretical basis of BDSP is divided into four levels. (i) The first level is the theoretical construction of the definition, connotation and attribution of BDSP, namely what is BDSP. (ii) The second level is the theoretical algorithm for the statistics, analysis and mining of BDSP, namely that classical algorithms are optimized to accommodate semantics types or new algorithms are developed for psychological characteristics. (iii) The third level is the theoretical practice for the information service of BDSP, namely the impact of psychological state of the worker on the risk evolution, which improves industrial facilities and work environments by psychological knowledge and rules. (iv) The fourth level is the theoretical innovation for the current challenge and future development trend of BDSP. The theoretical content of BDSP will inevitably change and adjust as the big data technology tends to mature.

#### Information Technology

Platform construction of big data mainly includes the module of data acquisition, storage, process and visualization. Every module is supported by its own hardware or software technology. The information technology of data acquisition, memory, processing and visualization will now be summarized as follows: (i) Currently, big data acquisition platforms include Apache Flume, Fluentd, Logstash, Chukwa, Scribe and Splunk Forwarder, which provide reliable and scalable data acquisition ways. (ii) The database system is a warehouse to organize, store and manage data, including relational database (e.g., Oracle, DB2, MySQL) and non-relational database (e.g., NoSql, Cloudant). Data warehouse, as one of the data integration frameworks, is an effective way to solve the problems of data analysis and to apply big data. (iii) Many data statistical and analytical techniques have emerged to solve the problem of massive data, data consistency, high concurrency and high availability in big data mining. Data algorithms should satisfy the technological and practical needs. (iv) Data visualization tools (e.g., Jupyter, Tableau, Google Chart, D3.js) will help users to identify future trends and patterns of psychological issues so as to make evidence-based safety decisions.

#### Application Range

The advantages of BDSP are to provide users with potential, worthwhile and practical information to solve safety psychological issues. Application range can be classified into individual, small-group, large-group and industrial levels according to the volume of study object and the requirement of information services (Kang et al., 2017b). Individual psychology refers to the psychological phenomena and behavioral pattern at the individual level, focusing on people’s psychological process, state and feature. Group psychology and industrial psychology focus on the psychological insight at the statistical level, which guides significance to intervene climates in safety activities. It should be noted that there is no clear-cut distinction between the small-group psychology and the large-group psychology. Users will obtain help from psychological insights, for example, (i) Individual BDSP provide the worker with an early warning service when they are faced with danger. (ii) Group BDSP provide the enterprise or department with feedback or correction services in safety education and safety management. (iii) Industrial BDSP provide a safety climate to intervene in the crisis incident.

## The Challenges of BDSP

With the growing amount of psychological related data, the good news is that the path is relatively straightforward to extract psychological information from big data, but the challenge lies in how to effectively capture, manage and extract them (Demystifying Big Data, 2012). Despite this, big data will help researchers to model, analyze and predict the psychological influence mechanism in the safety domain, some challenges still need to be outlined due to big data constraints.

### The Inherent Defects of Data

The inherent defects of data bring challenges to the development of BDSP:

(1) Data are obtained from the third-party data source in the present situation. The contents of those third-party data sometimes differ from each other, which may lead to invalidity and even faults in the mining results.
(2) The system has collected data that cannot completely reflect people’s psychological states due to the limitation of software input. In addition, multimodal, semantic analysis data needs to be considered. Semantic analysis techniques analyzing and categorizing psychological texts are relatively mature, with the support of the semantic dictionary (e.g., LIWC, Textmind), but the semantic analysis of video and audio is far from maturity.
(3) The deviation of personal characteristics has affected the representativeness of raw data (Kern et al., 2016), for example, some people hide their extraversion in the social network while they reveal an introversion characteristic in real life. Besides, online water armies, especially internet robots, can release false information by imitating human network activities, which decrease the reliability of the semantic analytic results.
(4) A large amount of data does not mean that they include the entire study objects (Weeg et al., 2015). The software presents an inevitable feature of users’ age level, such as young, middle-aged or aged, which will bring about a phenomenon of survivorship bias. In addition, it is ignored that phenomenon like the late report, the false report and the missing report exist during data collection, which will affect the reliability of case analysis methods.
(5) Under current circumstances, it is difficult to take on real-time collection and processing people’s psychological data on a large scale in the industrial field, especially in developing countries, since employers haven’t realized the responsibility of paying attention to employees’ psychological states.
(6) Traditional database methods are optimized for faster access and summarization of data users that they want to query. It has caused a situation where data satisfy this pattern rather than the opposite (Dhar, 2013), which is not able to discover new psychological knowledge and rules.

### The Insufficiency of BDSP

Big data of safety psychology cannot replace the traditional method of safety psychology at current stages. First, BDSP currently focuses on the theoretical aspects and is now at the initial development stage, unable to resolve all psychological problems of safety domain. BDSP has become an essential complement to traditional safety psychology. Moreover, the traditional method of safety psychology includes the experimental method, sampling statistics and case analysis, which are adequate to address safety psychological issue at a micro level. It can verify the accuracy of psychological knowledge excavated by big data to improve the reliability of the conclusion. Lastly, as a study hotspot of safety psychology, advantages and defects of BDSP will gradually emerge with deeper research and study as well as the development of information technology.

### The Ethical Issues of BDSP

Ethical issues of BDSP deserve serious consideration. Some studies have revealed that possible violations in big data are against user privacy (Barnaghi et al., 2013; Sivarajah et al., 2017). On the one hand, data collection and mining inevitably involves users’ private information, especially as users’ characteristics and habits are under analysis by big data, increasing the frequency of harassment services and causing privacy intrusions and invasive marketing. On the other hand, data are viewed as a commercial resource for organizations and not for the public. Unlicensed web crawlers will interfere with the normal running of the network system, and anti-crawling programs of system setup also affect the quality of data collected.

## Conclusion

Traditional safety psychology is not developed enough to solve human psychological problems in complex industrial production sometimes. Fortunately, there is an imminent need to convert such data into useful information and knowledge in industrial safety due to the wide availability of huge amounts of psychological data. Big data now becomes a new way to analyze people’s psychological states and behavior tendencies by processing a large amount of data. It is of great significance to discuss how big data is applied in the field of safety psychology. As an innovative approach aiming to excavate psychology related insight in industrial safety domain, BDSP offers some potential and valuable psychological knowledge and rules for safety practitioners to reduce the accident and to create a better working environment. In conclusion, BDSP significantly contributes to industrial safety and deserves thorough research by related scholars.

However, it is not enough for big data to be viewed as an important driving force to solve the psychological issues in the safety domain because it lacks supporting theories for guiding the application big data in safety psychology field. Under these circumstances, the main aim of this work is how to integrate big data into safety psychology and how to use it as a tool in the safety domain, providing the theoretical foundation of big data application in safety psychology field.

According to a literature review and comparative methods, we analyzed BDSP according to four aspects: (i) What BDSP are. First, the defect of the traditional study of safety psychology was analyzed and how big data extracts psychological rules were briefly reviewed. Then, the feasibility of BDSP was verified and the concept and aim of BDSP were proposed. Lastly, the differences between BDSP and traditional sample data were analyzed from 12 aspects. (ii) What the types and characteristics of BDSP are. First, seven classification standards of BDSP were analyzed to better understand its future development trends. Then, seven basic characteristics of BDSP were put forward according to the layer of dataset, technology and application. (iii) What the framework and structure of BDSP are. First, the framework of BDSP was constructed from four perspectives which are data requirements, study method, study tool, and processing step, which effectively manage the stream of big data. Then, a three-dimensional structure of BDSP was constructed at a theoretical level, information technology, and application range, by which its contents were clarified. (iv) What the challenges of BDSP are. It has outlined the challenge in terms of data inherent defect, BDSP insufficiency and ethical issues. These issues certainly require attention when applying big data in safety psychology research.

It is clear that big data plays an important role in the field of safety psychology. This study provides guidance to the evidence-based services in behavior management, which is valuable to help safety practitioners solve psychological issues in the aspects of safety management, safety education and safety enforcement.

## Data Availability

No datasets were generated or analyzed for this study.

## Author Contributions

LK contributed to the conception of the study. LK, CW, and BW contributed significantly to the manuscript preparation. LK wrote the manuscript. CW and BW helped to perform the analysis with constructive discussions.

## Conflict of Interest Statement

The authors declare that the research was conducted in the absence of any commercial or financial relationships that could be construed as a potential conflict of interest.
